# Restructuring and Hydrogen Evolution on Sub-Nanosized Pd_x_B_y_ Clusters

**DOI:** 10.3390/molecules29153549

**Published:** 2024-07-28

**Authors:** De Zhang, Ruijing Wang, Sijia Luo, Guangfeng Wei

**Affiliations:** Shanghai Key Laboratory of Chemical Assessment and Sustainability, School of Chemical Science and Engineering, Tongji University, Shanghai 200092, China

**Keywords:** palladium boride, cluster, hydrogen evolution reaction, DFT calculations

## Abstract

As a Pt-group element, Pd has been regarded as one of the alternatives to Pt-based catalysts for the hydrogen evolution reaction (HER). Herein, we performed density functional theory (DFT) computations to explore the most stable structures of Pd*_x_*B*_y_* (*x* = 6, 19, 44), revealed the in situ structural reconstruction of these clusters under acidic conditions, and evaluated their HER activity. We found that the presence of B can prevent underpotential hydrogen adsorption and activate the H atoms on the cluster surface for the HER. The theoretical calculations show that the reaction barrier for the HER on ~1 nm sized Pd_44_B_4_ can be as low as 0.36 eV, which is even lower than for the same-sized Pt and Pd_2_B nanoparticles. The ultra-high HER activity of sub-nanosized Pd*_x_*B*_y_* clusters makes them a potential new and efficient HER electro-catalyst. This study provides new ideas for evaluating and designing novel nanocatalysts based on the structural reconstruction of small-sized nanoparticles in the future.

## 1. Introduction

As a feasible hydrogen production method, water splitting is crucial to solve renewable energy and environmental problems [1,2]. Acidic water electrolysis via the hydrogen evolution reaction (HER, 2H^+^ + 2e^−^ → H_2_) is reported to have the highest energy efficiency and fastest kinetics for water splitting [3,4]. Currently, Pt nanoparticles are the main commercial catalyst for electrolysis because of their high HER activity and stability under acidic conditions [5,6]. However, due to the high price and low natural abundance of Pt [7], researchers are eager to find alternatives with higher activity and stability for Pt-based catalysts. As a Pt-group element, Pd has a similar hydrogen adsorption energy to Pt [8], which has become an important expectation for ultra-high-HER-activity catalysts. Nevertheless, the HER performance of pure Pd is inferior to pure Pt experimentally [8,9], which is affected by the catalytic site reconstruction caused by the formation of palladium hydride via underpotential hydrogen adsorption, according to previous reports [10,11,12].

To improve the activity and stability of Pd-based catalysts under acidic conditions, eliminating the underpotential hydrogen adsorption and activating the surface H atoms are necessary. There are three main strategies utilized in studies for this: (i) to form alloys with other metals, such as Pt [13,14] and Cu [15,16]; (ii) to dope main group elements, such as P [17,18] and N [19,20,21]; and (iii) to downsize to a cluster [22,23] or single atom [24,25]. Recently, a B-doped Pd_2_B nanosheet was reported to have a higher HER activity than Pt nanoparticles [26], indicating the possibility of designing ultra-high-activity Pd-based catalysts with the presence of boron [27]. Therefore, we consider whether the size effect of the clusters can be utilized to enhance the HER activity of palladium boride. On the other hand, for clusters, due to the surface effect, quantum size effect [28,29], and multi-bonding modes of boron [30], the effects of structure reconstruction on the catalytic activity of clusters becomes a complex problem under acidic conditions. Previous studies have shown that the HER activity of Pt group-based clusters, such as Pt [31], Ir [32], PdRu [33], and CoNi [34], is significantly affected by the type of exposed surface site and the dynamic structural evolution under reaction conditions. However, it is still a large challenge in the field of catalysis to artificially regulate the dynamic evolution behavior of nanoparticles and modulate their catalytic performance by changing the surface site distribution. The intrinsic relationship between particles’ composition and their structural reconstruction behavior urgently needs to be revealed.

Herein, we performed density functional theory (DFT) computations combined with the neural network stochastic surface walking (NN-SSW) global optimization method to explore the structure reconstruction of sub-nanosized Pd*_x_*B*_y_* (*x* = 6, 19, 44) clusters under HER conditions with different sizes and boron contents, and we evaluated their HER activity. To this end, a systematic step-by-step adsorption approach is presented that obtained the structure reconstruction under acidic conditions. We analyzed how the reconstruction was affected by the presence of B and H adsorption and found that increasing the B content and H content led to a more radical reconstruction of the Pd sub-structure. In our result, the migration of H atoms into the cluster sub-surface can be effectively inhibited, and H atoms on the cluster surface can be activated by the presence of B, which improves the HER activity. We found Pd_44_B_4_ to have the lowest free-energy barrier (0.36 eV) for HERs, lower than Pt nanoparticles (0.95 eV) and Pd_2_B bulk (0.49 eV). Furthermore, we utilized partial wave density of state (PDOS) calculations to explain the effects of B atoms on the clusters’ HER activity.

## 2. Results and Discussion

### 2.1. The Structures of Sub-Nanosized Pd_x_B_y_ Clusters

**Figure 1:**
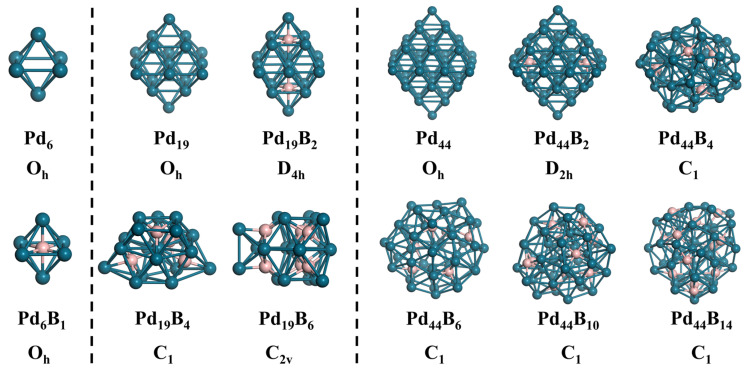
The most stable structures for Pd*_x_*B*_y_* (*x* = 6, 19, 44) acquired using NN-SSW and checked by DFT.

The stochastic surface walking (SSW) global structural optimization method was employed to find the most stable structures (global minima, GM) of Pd*_x_*B*_y_* clusters with varying sizes and B contents. These structures are illustrated in Figure 1. The GM structures of the Pd clusters exhibit a highly symmetric (O_h_) arrangement, with all Pd atoms following the face-centered cubic (*fcc*) packing mode observed in bulk Pd. However, the structures of the Pd*_x_*B*_y_* clusters can significantly deviate from those of bulk Pd, even when a small amount of B is present. The symmetry of the Pd*_x_* clusters is preserved only in the smallest Pd_6_B cluster and the other Pd*_x_*B*_y_* clusters with very low B content. In these clusters, the B atom occupies the O_h_ interstitial site, forming an octahedral [Pd_6_B] structural unit. Remarkably, this [Pd_6_B] unit corresponds precisely to the basic structural unit of bulk Pd_2_B boride [26], which is known to exhibit excellent intrinsic activity for the hydrogen evolution reaction (HER). For the Pd_19_ cluster, we found Pd_19_B_2_ (B content: 9.5%) retains most of the original structure of Pd_19_. Meanwhile, the B atoms still form an octahedron [Pd_6_B] structure unit, although the presence of B atoms causes lattice expansion and local symmetry breaking. However, with an increasing B content, the structures of Pd_19_B_4_ and Pd_19_B_6_ are quite different from Pd_44_ with the B atoms forming additional low coordination structure units (illustrated in Figure S2). In increasing the cluster size to Pd_44_, the structure of Pd_44_B_2_ (with a B content of 4.3%) closely resembles the Pd_44_ global minimum (GM) structure. However, this inserted subsurface B resulted in lattice expansion and distortion on the apexes of the Pd_44_ octahedron. Unlike Pd_19_, Pd_44_ exhibits a core–shell structure, leading to more complex structural reconstruction. In GM structures, B atoms tend to reside between the core and shell or on the cluster surface, while the core structure remains less affected. Interestingly, the core structures of clusters with varying B content exhibit striking similarity in their topology (as shown in Figure S4a), despite an increase in the number of Pd atoms in the core (from 7 to 8). This behavior aligns with the “hard core and soft shell” phenomenon observed in Pt clusters [31], which can be attributed to the higher stability of the atoms in the core region and smaller energy difference between the structures with the same core structure. The stability of these Pd*_x_*B*_y_* clusters can be verified via their relative formation energy (see Figure S1). Compared to the pure Pd cluster and bulk B (*α*-phase), the formation of Pd*_x_*B*_y_* clusters is all thermodynamically exothermic, which implies that the B insertion is an irreversible process.

Summarizing the structure evolution of the Pd*_x_*B*_y_* clusters induced by the increased B atoms, there are three aspects: (i) Increasing the B content in a Pd*_x_*B*_y_* cluster can lead to reconstruction from the pure Pd clusters’ octahedron structure to a quasi-spherical structure. (ii) The diversity of B bonding and the increasing B content result in the emergence of various structure units composed of B and Pd atoms. These units contribute to achieving more stable cluster structures. (iii) Different B content and structure units primarily affect the shell structure of clusters, while their influence on the core structure of the cluster is relatively small. Clearly, due to the structural reconstruction features of Pd*_x_*B*_y_* clusters, elucidating their true structures under reaction conditions is particularly important for understanding their HER performance.

### 2.2. Structure Analysis of Pd_x_B_y_H_z_ under HER Conditions

**Figure 2:**
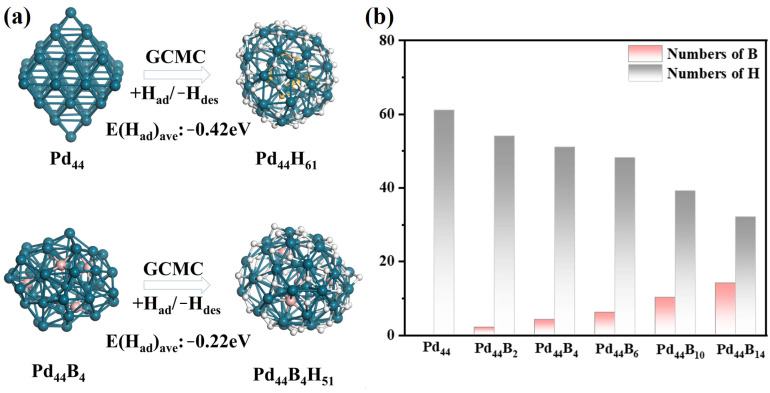
The adsorbed H atoms on Pd*_x_*B*_y_* clusters. (**a**) The reconstructed structures of Pd_44_ and Pd_44_B_4_ clusters under HER conditions (Orange atoms are H inside the cluster, white atoms are H on the cluster surface). (**b**) The trends in the number of B and H atoms in the reconstructed structures for GM Pd_44_B_x_H_z_ clusters.

To obtain the reconstructed structures of Pd*_x_*B*_y_*H*_z_* clusters under the HER environment, we applied grand canonical Monte Carlo (GCMC) simulations via dynamically adding or removing H atoms (see calculation details). In the simulations, the partial pressure of hydrogen was maintained at 1 atmospheric pressure. As shown in Figure 2a, under HER conditions, Pd_44_ clusters can adsorb up to 61 H atoms, which resulted in a large reconstruction of its structure.

**Figure 3:**
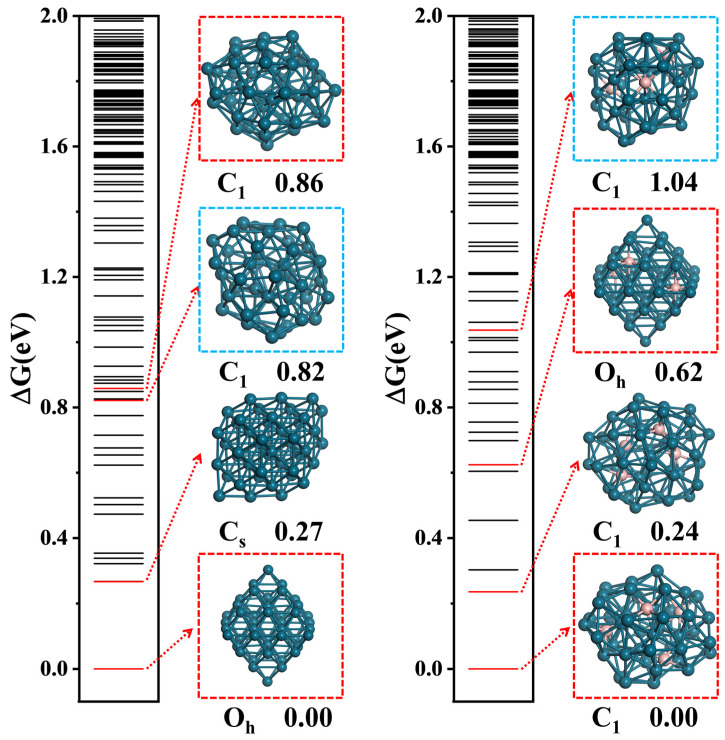
Low-lying isomer diagrams of Pd_44_ and Pd_44_B_4_ (the blue frame is the Pd sub-structure under HER conditions, and the red frame is the similar Pd sub-structures with the difference of B atoms).

The GM structure of Pd_44_H_61_ shifted from the highly symmetrical O_h_ structure to a quasi-spherical C_1_ structure. The average bond length of the surface Pd-Pd bond increased from 2.72 Å to 2.81 Å due to the bonding of H atoms, resulting in the reconstruction of the overall lattice expansion. In addition, a Pd atom migrated from the surface to the core, which changed the original O_h_ symmetry core structure into a D_5h_ core structure during the reconstruction (core atoms from 6 to 7, see Figure S3). In the equilibrium structure of Pd_44_H_61_, most of the adsorbed H atoms sat on the surface three-fold hollow sites and the two-fold edge sites, while eight of the adsorbed H atoms imbedded into the subsurface layer of the cluster. These subsurface H atoms occupied the interstitial site of three Pd atoms, forming a [Pd_3_H] unit. The average H adsorption energy was −0.42 eV, in the low-lying isomers of Pd_44_, which can be found in Figure 3. The energy of the Pd sub-structure of Pd_44_H_61_ (via removing all the H atoms) was 0.82 eV higher than the most stable O_h_ Pd_44_. Considering the larger H adsorption amount, the energy cost of the reconstruction was much lower than the total H adsorption energy. This result indicates the occurrence of structure reconstruction is very possible under HER conditions, and H atoms will imbed into the subsurface layer. This is consistent with the underpotential hydrogen adsorption reported in the experiment.

With the presence of B, the cluster structure was further reconstructed under HER conditions. We noticed that the adsorbed H atoms decreased uniformly with the increasing B content, as presented in Figure 2b. Interestingly, only a small amount of B can prevent H atoms from migrating into the inner cluster. At Pd_44_B_4_, shown in Figure 2a, the H atoms completely adsorbed on the cluster surface, although only three H atoms were in the inner cluster at Pd_44_B_2_. These H atoms will not bond with B atoms in the cluster and still occupy interstitial sites or edge sites composed of Pd atoms. The repulsion of the H atom and B atom can be considered to be one of the reasons for the decrease in the H adsorption quantity, and it leads to a decrease in the H adsorption energy too. In Pd_44_B_4_, the average adsorption energy was −0.22 eV, which was much lower than Pd_44_. With the increase in the B content, the average adsorption energy of H continuously decreased (to −0.09 eV) and showed good linearity (see Figure S5). However, although the average H adsorption decreased after the presence of B, the total H adsorption energy could overcome the energy barrier of cluster reconstruction under HER conditions. We can compare the energy of the reconstructed Pd sub-structure for Pd_44_B_4_, which was 1.04 eV higher than before, as shown in Figure 3, and this energy for the other clusters was 0.58~1.83 eV (see Figures S6–S13), which was far lower than the total H adsorption energy.

By comparing the low-lying isomer diagrams of Pd_44_ and Pd_44_B_4_, we can also see that the reconstruction of clusters caused by hydrogen adsorption is similar but slightly different from that caused by the presence of B. This can be discussed in four points. (i) The energy difference in the Pd sub-structure during reconstruction caused by hydrogen adsorption and the presence of B is similar. The Pd sub-structure of Pd_44_H_61_ is 0.82 eV, shown in Figure 3, which is very close to the 0.82 eV in the Pd sub-structure of Pd_44_B_4_. (ii) The reconstruction of highly symmetric structures to approximately spherical structures is a thermodynamically stable process no matter the hydrogen adsorption or presence of B. In Pd_44_B_4_, we found the local minimum with an O_h_ symmetric Pd sub-structure, its energy was 0.62 eV higher than GM, and this energy increase still existed in other clusters with different B content. (iii) The increasing B content decreases the adsorption capacity and average H adsorption energy and prevents H from imbedding into the cluster subsurface layer. (iv) The adsorption of H has little effect on the structural units of surface B atoms but has a higher effect on the core structure of the clusters (see Figure S4b). Hydrogen adsorption may lead to the migration of one B atom from the cluster subsurface to the core, forming relatively similar core structures of all the clusters. In order to further explore the effects of these reconstructions on the HER activity of the whole cluster, DFT calculations were used to evaluate the HER free-energy barrier of each cluster at the best catalytic site.

### 2.3. Free-Energy Barrier of the HER and the Catalytic Unit

**Figure 4:**
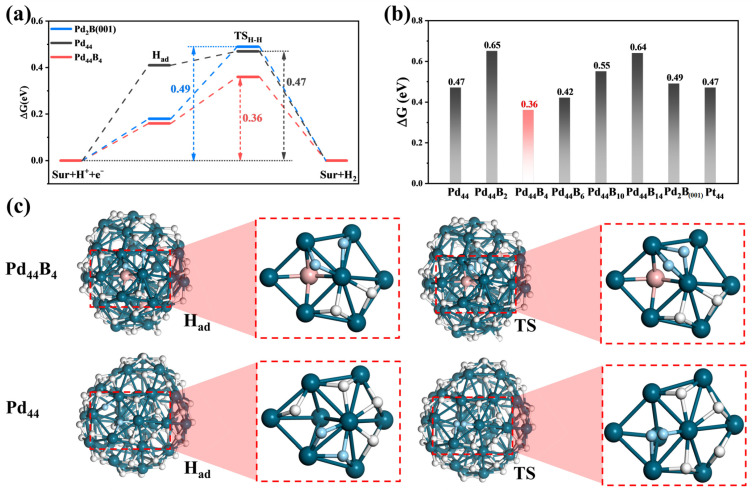
The HER performance evaluation for the best Pd_44_B_4_ cluster. (**a**) The reaction profiles for the HER process on Pd_2_B (001), Pd_44_, and Pd_44_B_4_ at U = 0 V vs. SHE. (**b**) The energy barriers of the HER on Pd_44_B*y* (*y* = 0, 2, 4, 6, 10, 14), Pd_2_B (001), and Pt_44_. (**c**) The structural snapshots with vital catalytic units of the H_ad_ and TS states on the Pd_44_ and Pd_44_B_4_ clusters (the white atoms are the adsorbed unreacted H, and the light blue atoms are the reacting H).

Considering that the selection of catalytic sites is particularly important for clusters, we had to select the best catalytic site, which was the hydrogen molecule adsorbed site, when the HER occurred during GCMC. In this process, the differential adsorption energy (DHG) of the additional H atom was slightly higher than 0 eV. We calculated the HER activity of all the clusters in Figure 1 at the best catalytic site selected. The Tafel mechanism of the surface H coupling reaction H* + H* → H_2_ is known to be the major pathway for the HER on active metals. Then, we compared the HER activity of Pd_44_B_4_ (our best), Pd_44,_ and Pd_2_B (001) for this mechanism in Figure 4a.

To study the Tafel reaction of the HER, one first needs to know the equilibrium surface H coverage under reaction conditions, where the adsorbed H equilibrates with the solvated protons in the electrolyte. This was determined via the DGH during the GCMG process using the DFT calculations, as before. Next, we examined the kinetics of two surface H coupling to form a H_2_ molecule on these cluster surfaces. Figure 4a,b shows the HER free-energy barriers (∆G) of Pd_44_B_4_, Pd_44_, and Pd_2_B (001) and the ∆G changes of Pd_44_B_y_ series. Figure 4c shows the adsorbed state of the additional H atom (H_ad_) structures and transition state (TS) structures of Pd_44_B_4_ and Pd44 during the HER process and the comparison of important catalytic units in H_ad_ and TS. The Volmer step occurs first with one proton from the solution adsorbing on the surface with the simultaneous electron transfer: * + H^+^ + e^−^ → H*. Next, this additional H reacts with the nearby surface H to achieve the TS, which is a Tafel step: H* + H* → H_2_. The TS is a [H-H] complex near a top site with the H-H distance in the range of 0.80–1.40 Å. Despite having the same reaction mechanism, the best catalytic sites and ∆G for these clusters are markedly different. As shown in Figure 4a, the ∆G of Pd_44_ for the HER is 0.47 eV. What is more, when B atoms are incorporated to form Pd_44_B_4_, the ∆G decreases to 0.36 eV, which is 0.13 eV lower than the previous best Pd_2_B (001) and 0.11 eV lower than the best HER top site of Pt_44_. It is worth noting that there is a nonlinear relationship between the ∆G and the boron content, as shown in Figure 4b. We found that when two boron atoms were incorporated to form Pd_44_B_2_, the energy barrier increased slightly to 0.65 eV, while increasing the B content to form Pd_44_B_4_, the ∆G decreased significantly to 0.36 eV, and then, in continuing to increase the B content (from Pd_44_B_4_ to Pd_44_B_14_), ∆G gradually increased to 0.58 eV.

In order to understand this phenomenon, we analyzed the structures of Sur, H_ad_, TS, and H_2ad_ during the HER process and the catalytic units of each cluster (see Figures S14–S18), and selected Pd_44_ and Pd_44_B_4_, as shown in Figure 4c, as a comparison. We found that the catalytic process and catalytic unit were similar; the catalytic atom was located in a planar tetragonal structure composed of four Pd atoms. However, starting from Pd_44_B_4_, there was a B atom under the planar tetragonal structure, which is an important factor to reduce the ∆G (this unit also exists in Pd_19_B_y_, see Figure S16). This B atom leads to the decrease in the number of H atoms adsorbed on the catalytic unit and the activation of surface H, where an external H atom is used to form the [H−H] complex (1.19 Å) at the bridge site. However, although there is a similar catalytic site and HER mechanism in Pd_44_, the surface H atom is not activated due to the absence of B, which leads to a higher energy barrier. On the other hand, the continuous increase in the B content causes the proportion increase in the surface B atoms. This induces a higher energy barrier of additional adsorbed hydrogen atoms on the surface. The decrease in the surface H coverage causes an increase in the energy required to reach the TS, thereby suppressing the catalytic HER activity.

## 3. Discussion

**Figure 5:**
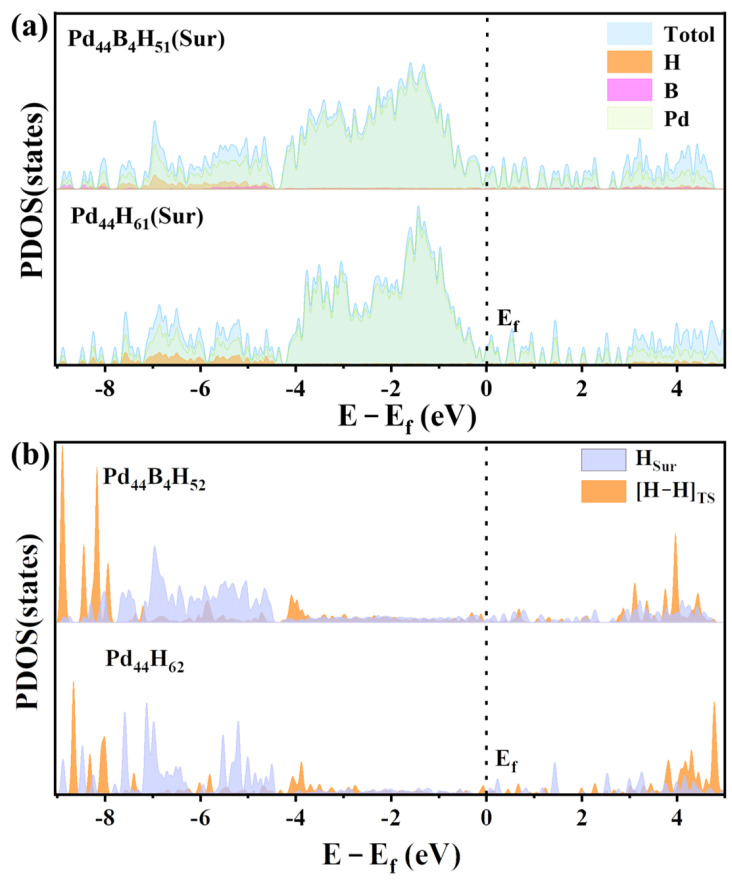
The partial wave density of states (PDOS) of vital HER states on Pd_44_ and Pd_44_B_4_ under the HER condition. (**a**) The PDOS of Pd, B, and H atoms on the Pd_44_B_4_H_51_ and Pd_44_H_61_ Sur states. (**b**) The PDOD of the surface adsorbed H (H_Sur_), reacting H ([H−H]_TS_) on the Pd_44_B_4_H_52_ and Pd_44_H_62_ transition states.

For the Pd*_x_*B*_y_* cluster, the proper B content (about 10%) can improve the HER catalytic activity. The projected density of states (PDOSs) was plotted to further explore the effects of the B atoms on the HER activity, presented in Figure 5a and Figure S19 for the Sur state and Had state, respectively. It can be found that Pd_44_ and Pd_44_B_4_ all have electron density near Fermi energy under HER conditions, which indicates they can conduct for HER. In the range of −4.0 eV~0.0 eV, it is mainly the d-orbital electrons of Pd, which means that the reduction electrons mainly come from the d electrons of Pd. The s electron of H overlaps with the p electron of Pd mainly at −7.0 eV~−4.5 eV and overlaps with the s electron of Pd mainly at −9.0 eV~−7.0 eV. The p electrons of B mainly overlap with the Pd p electrons at −6.0 eV~−4.5 eV and also overlap with the H s electrons at −6.0 eV~−4.5 eV. The lowest energy is the overlap of the s electrons of H, B, and Pd, and the energy is −9.0 eV~−8.0 eV.

In Figure 5b, we can see the electronic structure of the initial state (Sur) and the transition state (TS) of H in the HER. We can find that the electronic structure of H in the two clusters is similar at TS, but the electron density of H in the Pd_44_B_4_ cluster is significantly higher than Pd_44_ cluster (−4.5 eV~−6.0 eV) at Sur state. This energy segment is highly coincident with the overlapping energy segment of the p electrons of B and s electrons of H, which indicates that the reaction H on Sur is activated by B, resulting in the reduction in the energy barrier.

## 4. Calculation Methods

### 4.1. Stochastic Surface Walking (SSW) Sampling Method

We used the basic structure search module of NN-SSW [35] to obtain the most stable structures (global minima, GM) of the initial Pd*_x_*B*_y_* (*x* = 6, 19, 44) clusters by selecting more than 30,000 minima from more than 100 parallels at 600 K, performed in the LASP (www.lasphub.com, accessed on 1 January 2024) program. The Metropolis Monte Carlo scheme [31] was utilized to accept or reject a newly found structure in every SSW step. In this study, the Gaussian width (ds) was set as 0.6, and the Gaussian number was 10.

To intensively explore the structure reconstruction of Pd*_x_*B*_y_*(*x* = 6, 19, 44) clusters under HER conditions, grand canonical Monte Carlo (GCMC) simulations combined with SSW optimization were applied. During the GCMC simulations, a pair of hydrogen atoms was stepwise added into or removed from the system; the adsorption configuration was determined by the SSW global optimization. The partial pressure of hydrogen was maintained at 1 atmospheric pressure. For each adsorption configuration, at least 3000 distinguished minima were collected from 10 parallels by SSW.

### 4.2. Density Functional Theory (DFT) Calculations

The projector-augmented-wave method (PAW) [36,37] was used in the DFT calculations, as implemented in the Vienna ab initio simulation package [38] (VASP 6.2.1) contained in the LASP program. The Monkhorst–Pack scheme with a k-point separation length of 0.05 Å^−1^ was utilized for sampling the first Brillion zone [39], which was set as 1 × 1 × 1, and the energy cutoff was set as 400 eV in all cluster calculations. We performed DFT calculations for all the GMs (including in the HER process) and the low-lying isomers of Pd*_x_*B*_y_*.

The free-energy barrier of the HER (∆G) is the standard to measure catalytic activity:

(1) $$∆G={∆E}_{H}+{∆E}_{ZPE}-T∆S$$

where T is the system temperature, and ${∆E}_{ZPE}$ and ∆S are the changes in zero-point energy and entropy. ${∆E}_{H}$ consists of one additional H atom’s adsorption energy (${∆E}_{ad}$) and the energy difference (${∆E}_{T})$ between the transition state (TS) and hydrogen adsorption state (H_ad_):

(2) $${∆E}_{H}={∆E}_{ad}+{∆E}_{T}$$

The free energy of a proton coupled with an electron (H^+^ + e^−^) can be regarded as 1/2$G_{H_{2}}$ under standard conditions. The TSs of the catalytic reaction were searched using the double-ended surface walking method [40,41,42]. The exchange–correlation functional utilized was at the generalized gradient approximation level with the Perdew–Burke–Ernzerhof (PBE) functional, known as the GGA-PBE [43]. To correct the zero-point energy for the reaction barrier, the vibrational frequency calculations were performed using the finite-difference approach.

## 5. Conclusions

In summary, this work elucidates the structure evolution of different sized Pd*_x_*B*_y_* clusters as well as their performance for the catalytic HER. The impact of the B presence and hydrogen adsorption on structure reconstruction can be categorized into three main aspects: (i) Both B and hydrogen induce substantial structural changes in the cluster, leading it toward an approximately spherical shape. This effect becomes more pronounced with increasing B content. (ii) The presence of subsurface boron can prevent the excessive hydrogenation of small Pd clusters, which is beneficial to the HER performance. (iii) Pd_44_B*_y_* clusters exhibit similar core topological structures in the presence of both boron and hydrogen adsorption. This similarity may enhance their stability under acidic conditions. By investigating the HER activity of different Pd*_x_*B*_y_* clusters, the cluster of Pd_44_B_4_ was predicted to have the highest HER activity, with the reaction barrier (only 0.36 eV) significantly lower than the pure Pt and Pd_2_B catalysts. The optimal B content for HER lies around 10% in Pd clusters, with a cluster size of approximately 1.1 nm (Pd_44_ series). This finding has critical implications for experimental synthesis, guiding the search for ultra-high HER catalysts to replace Pt metal.

## Data Availability

The authors confirm that the data supporting the findings of this study are available within the article and/or its Supplementary Materials.

## Supplementary Materials

The following supporting information can be downloaded at: https://www.mdpi.com/article/10.3390/molecules29153549/s1, Figure S1: The decrease in the GM energy (∆E) of the most stable cluster varies with the number of B atoms; Figure S2: Coordination geometry of B atoms in Pd*_x_*B*_y_* GM clusters; Figure S3: GM structures of Pd*_x_*B*_y_*H*_z_* clusters under HER conditions with their symmetry of Pd sub-structure; Figure S4: Changes in the core structures in Pd_44_B_y_ clusters with and without HER conditions; Figure S5: The change in the average H adsorption free energy (AHG) of Pd_44_B_y_H_z_ GM structures with the number of B atoms; Figures S6–S13: Low-lying isomers diagram of other Pd*_x_*B*_y_* clusters; Figures S14–S17: HER performance evaluation profiles with structural snapshots for Pd6 and Pd19 series; Figure S18: Structural snapshots of the HER occurrence for other Pd_44_B_y_ (y = 2,6,10,14) clusters; Figure S19: PDOS of Pd_44_B_4_H_52_ and Pd_44_H_62_ Had state for HER.
