# Supplementary material for: Restructuring and Hydrogen Evolution on Sub-Nanosized PdxBy Clusters
Source: Molecules. 2024 Jul 28;29(15):3549. doi: 10.3390/molecules29153549 (PMC11314066; doi:10.3390/molecules29153549)
Supplement: Supplementary file 1 [file molecules-29-03549-s001.zip › molecules-3105786-supplementary.pdf]

Restructuring and Hydrogen Evolution on Sub-nanosized Pd_xB_y Clusters

Supplementary Materials

De Zhang, Ruijing Wang, Sijia Luo and Guangfeng Wei *

Shanghai Key Laboratory of Chemical Assessment and Sustainability, School of Chemical Science and Engineering, Tongji University, Shanghai 200092, China.
* Correspondence: weigf@tongji.edu.cn

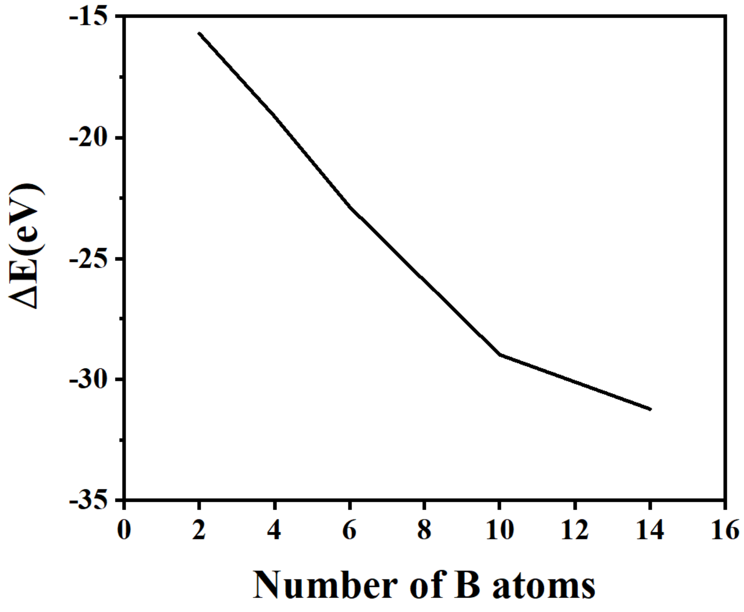

Figure S1. The decrease of GM energy (ΔE) of the most stable cluster varies with the number of B atoms.

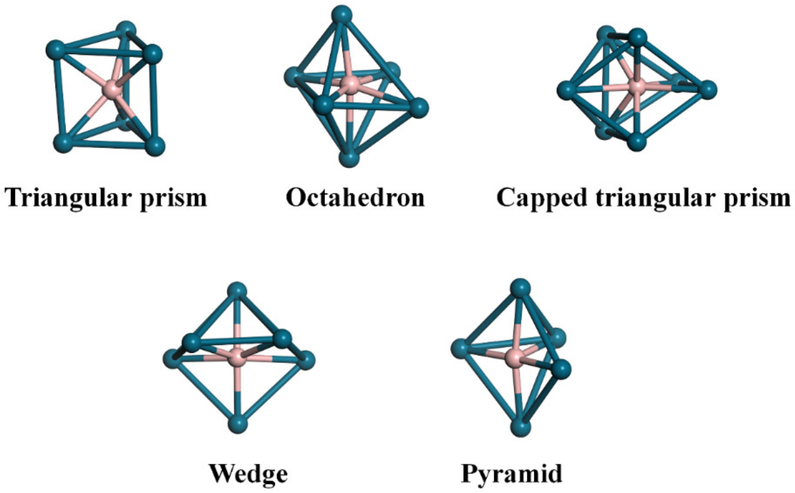

Figure S2. Coordination geometry of B atoms in Pd_xB_y GM clusters (deep-blue ball presents Pd atom, pink ball presents B atom).

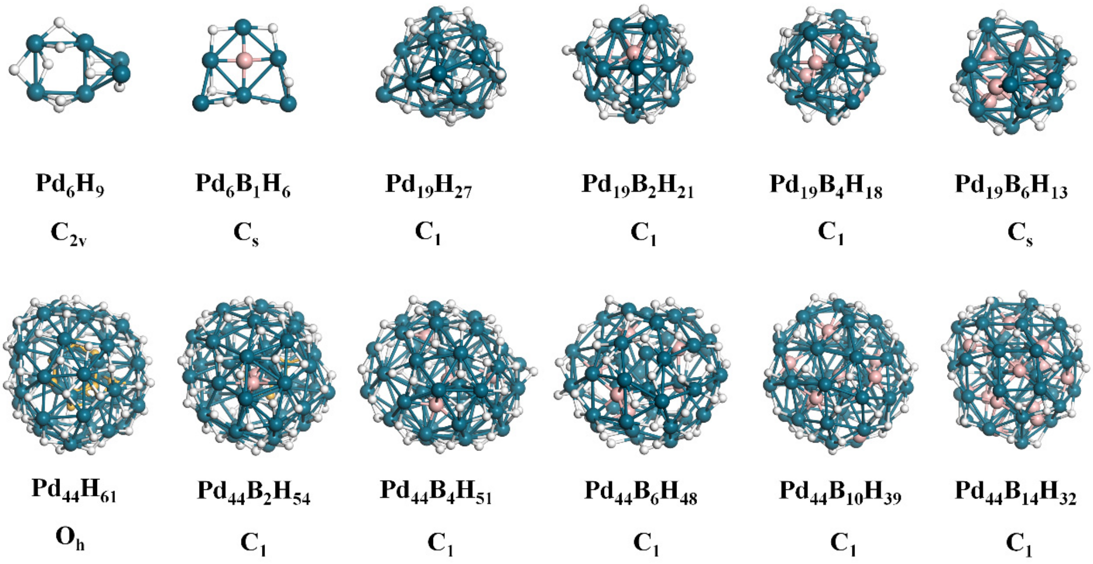

Figure S3. GM structures of $Pd_xB_yH_z$ clusters under HER conditions with their symmetry of Pd sub-structure (deep-blue ball presents Pd atom, pink ball presents B atom, white ball presents H atom on cluster surface, and orange ball presents H atom siting inner of the cluster).

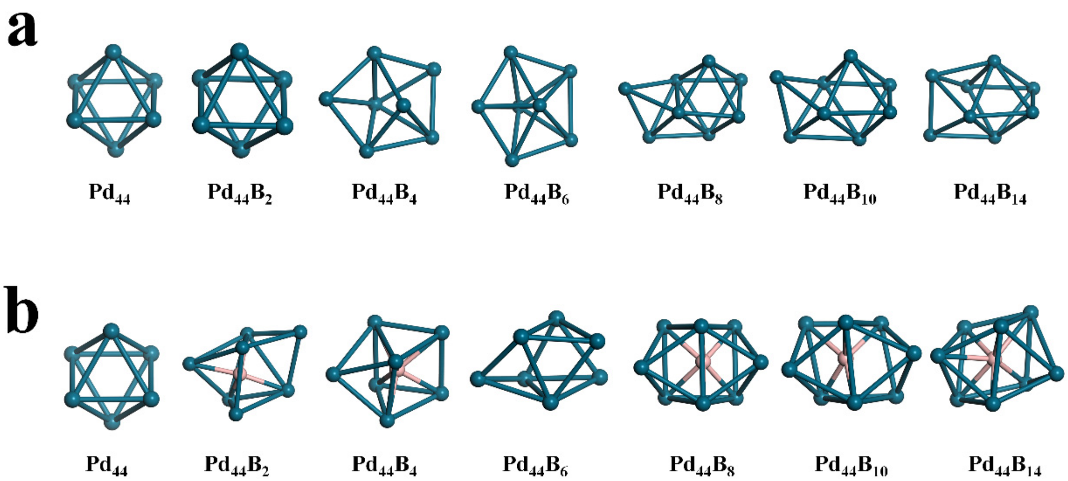

Figure S4. Changes of core structures in $Pd_{44}B_y$ clusters. (a) The core structures in Pd_xB_y clusters. (b) The core structures in $Pd_xB_yH_z$ clusters under HER conditions.

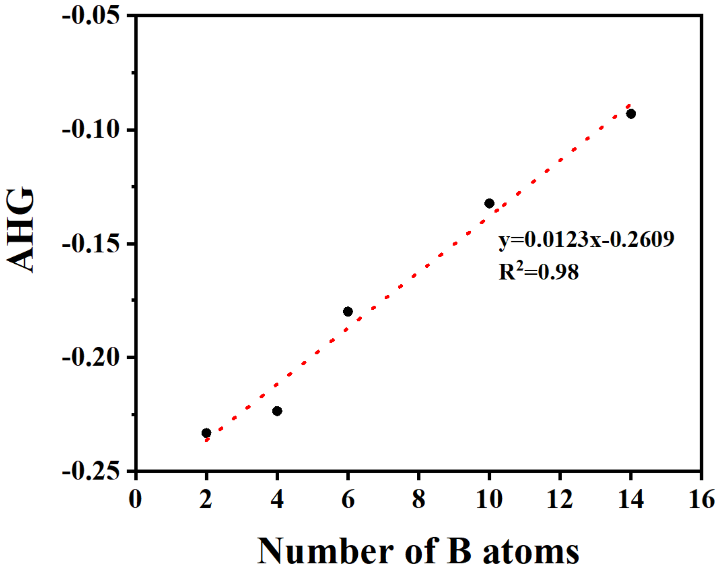

Figure S5. The change of average H adsorption free energy (AHG) of $Pd_{44}B_yH_z$ GM structures with the number of B atoms, and red line is its linear fitting.

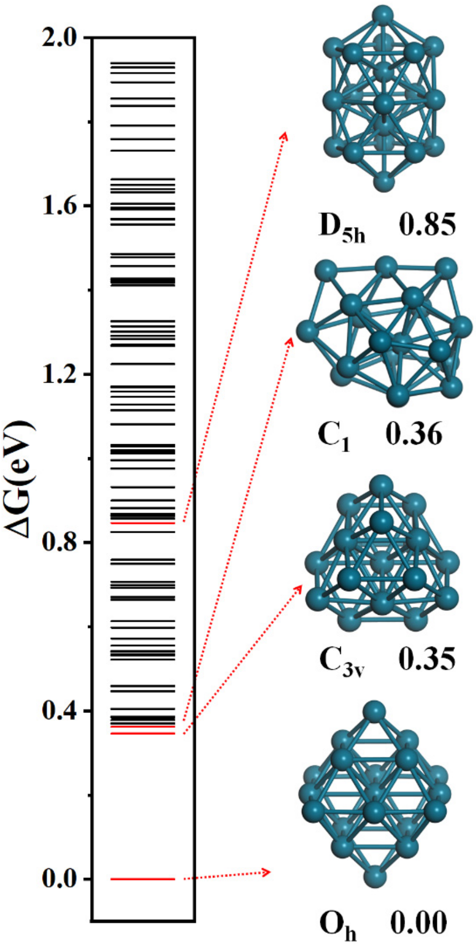

Figure S6. Low-lying isomers diagram of Pd_{19} clusters.

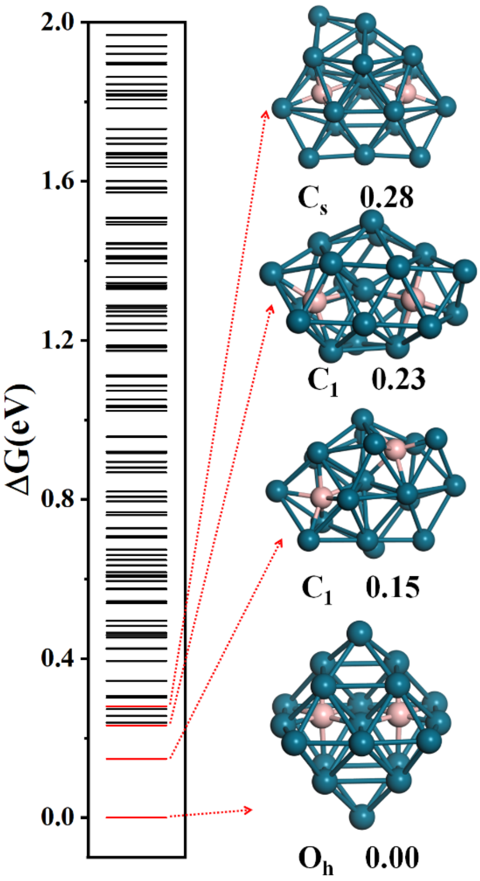

Figure S7. Low-lying isomers diagram of $Pd_{19}B_2$ clusters.

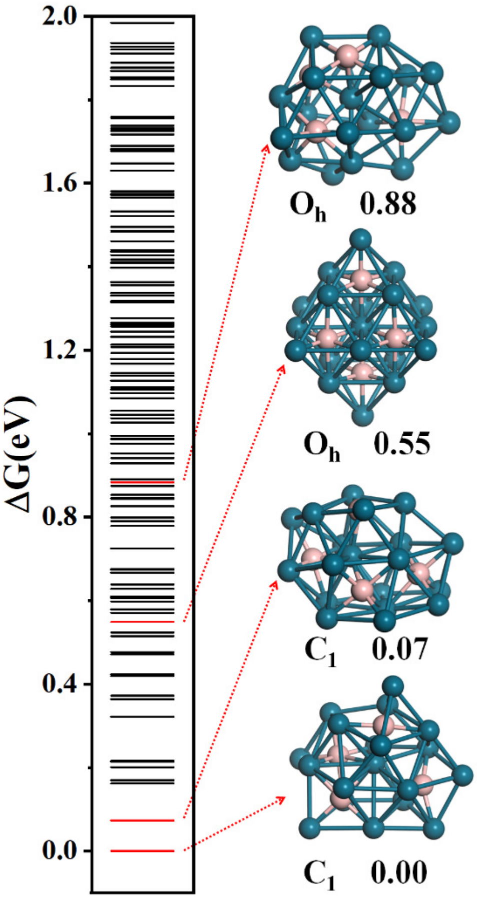

Figure S8. Low-lying isomers diagram of $Pd_{19}B_4$ clusters.

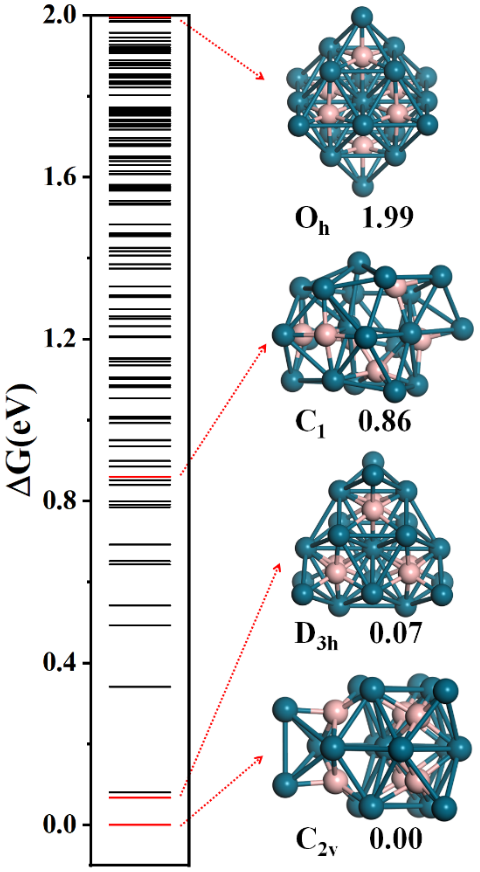

Figure S9. Low-lying isomers diagram of $Pd_{19}B_6$ clusters.

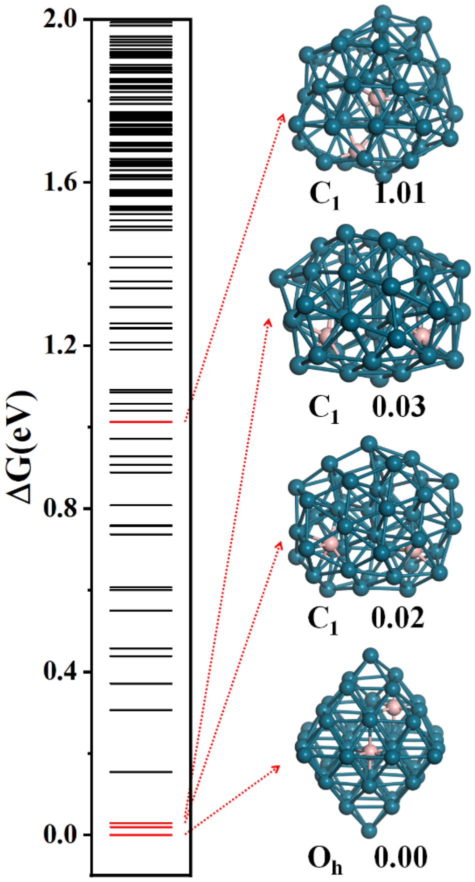

Figure S10. Low-lying isomers diagram of $Pd_{44}B_2$ clusters.

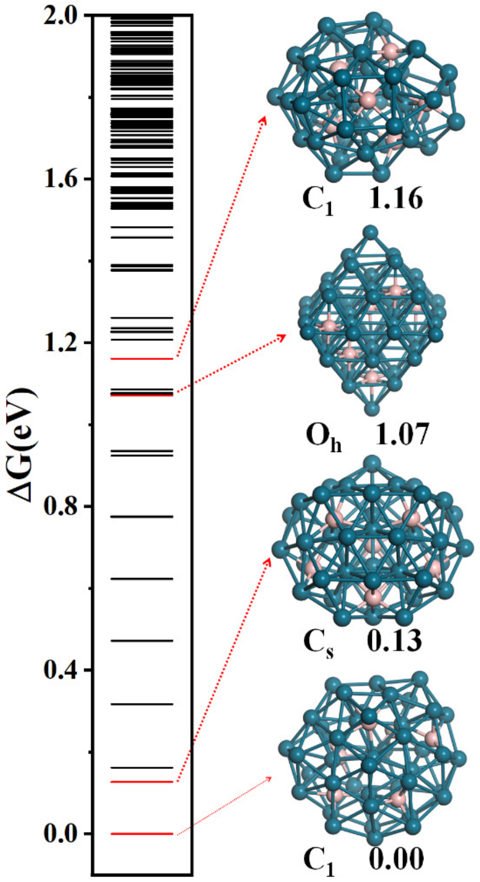

Figure S11. Low-lying isomers diagram of $Pd_{44}B_6$ clusters.

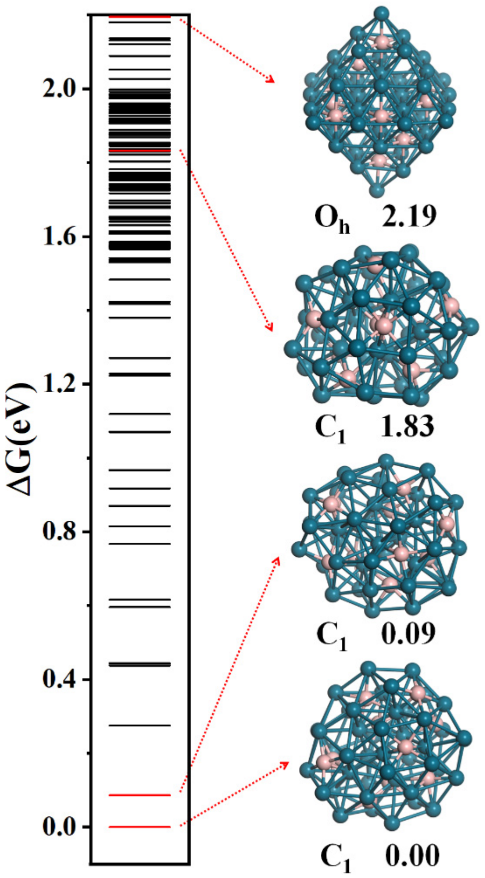

Figure S12. Low-lying isomers diagram of $Pd_{44}B_{10}$ clusters.

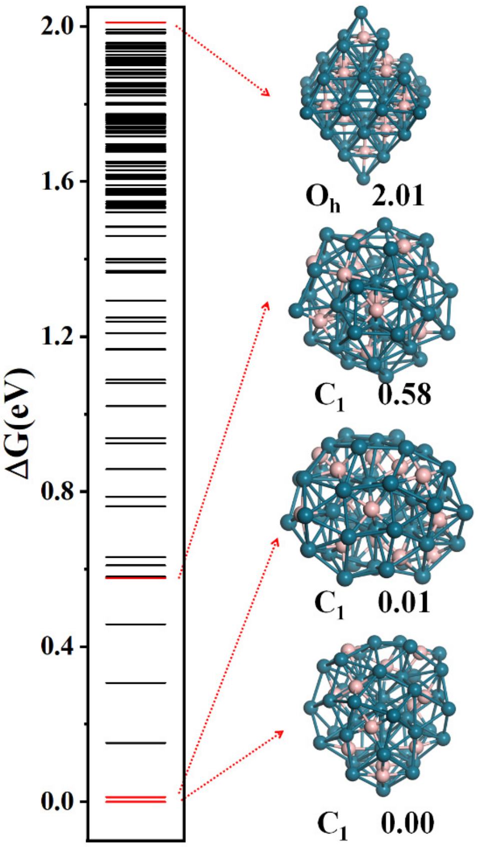

Figure S13. Low-lying isomers diagram of $Pd_{44}B_{14}$ clusters.

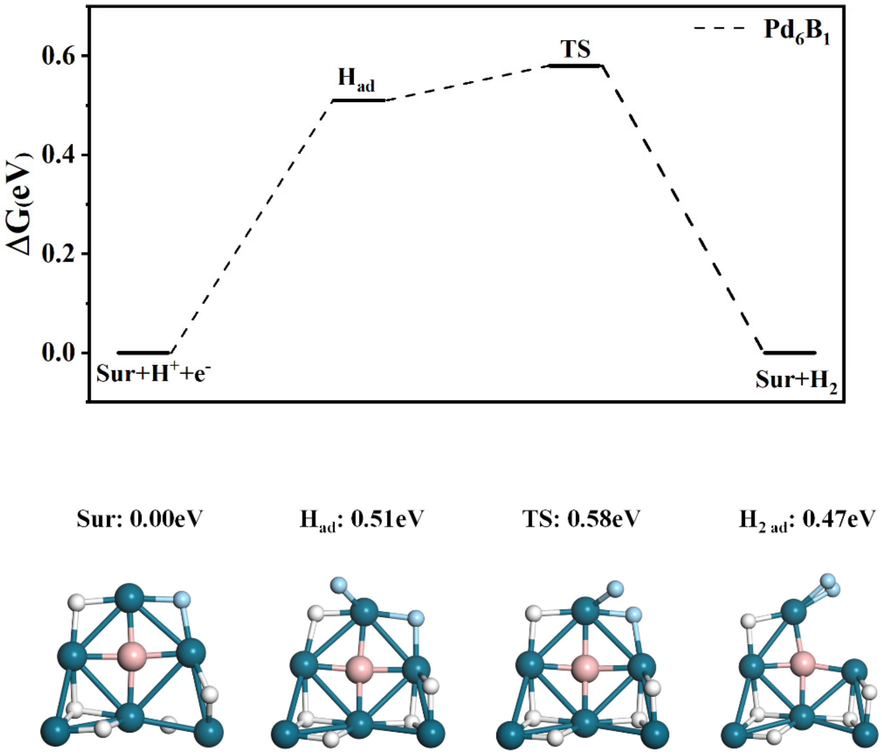

Figure S14. The HER energy diagram of Pd_6B cluster, and structures of every step during HER pathway.

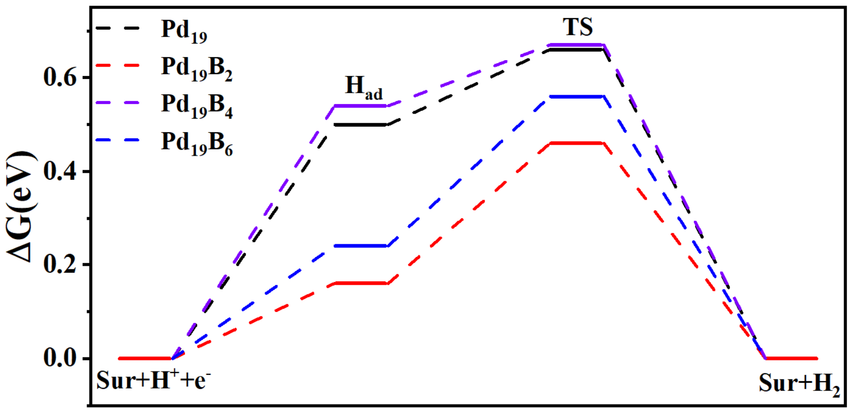

Figure S15. The HER energy diagram of $Pd_{19}B_y$ (y=0,2,4,6) clusters.

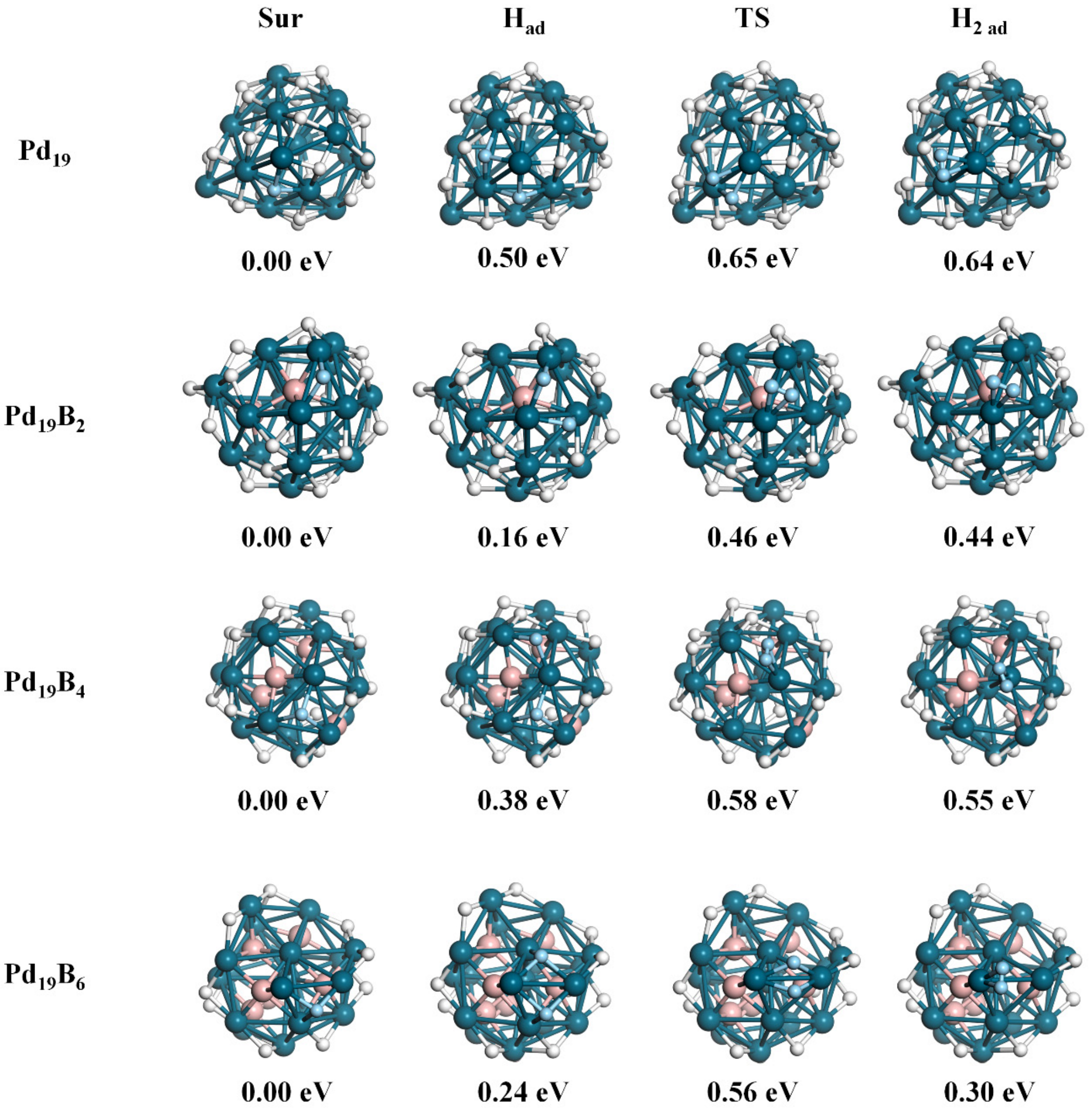

Figure S16. The structures of every step during HER pathway for $Pd_{19}B_y$ (y=0,2,4,6) clusters.

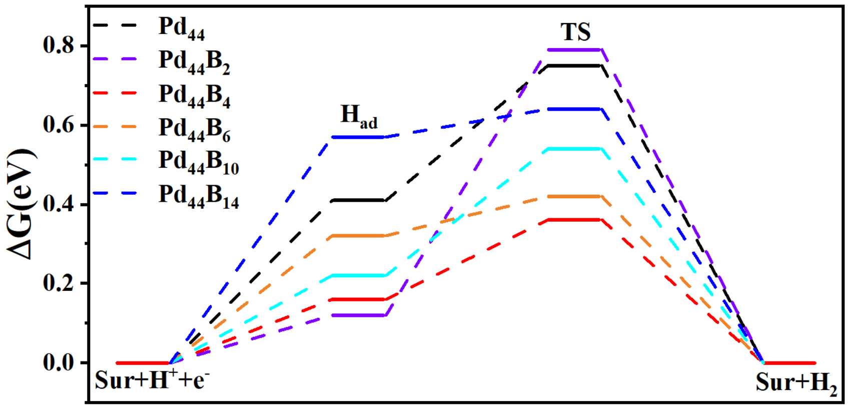

Figure S17. The HER energy diagram of $Pd_{44}B_y$ (y=0,2,4,6,10,14) clusters.

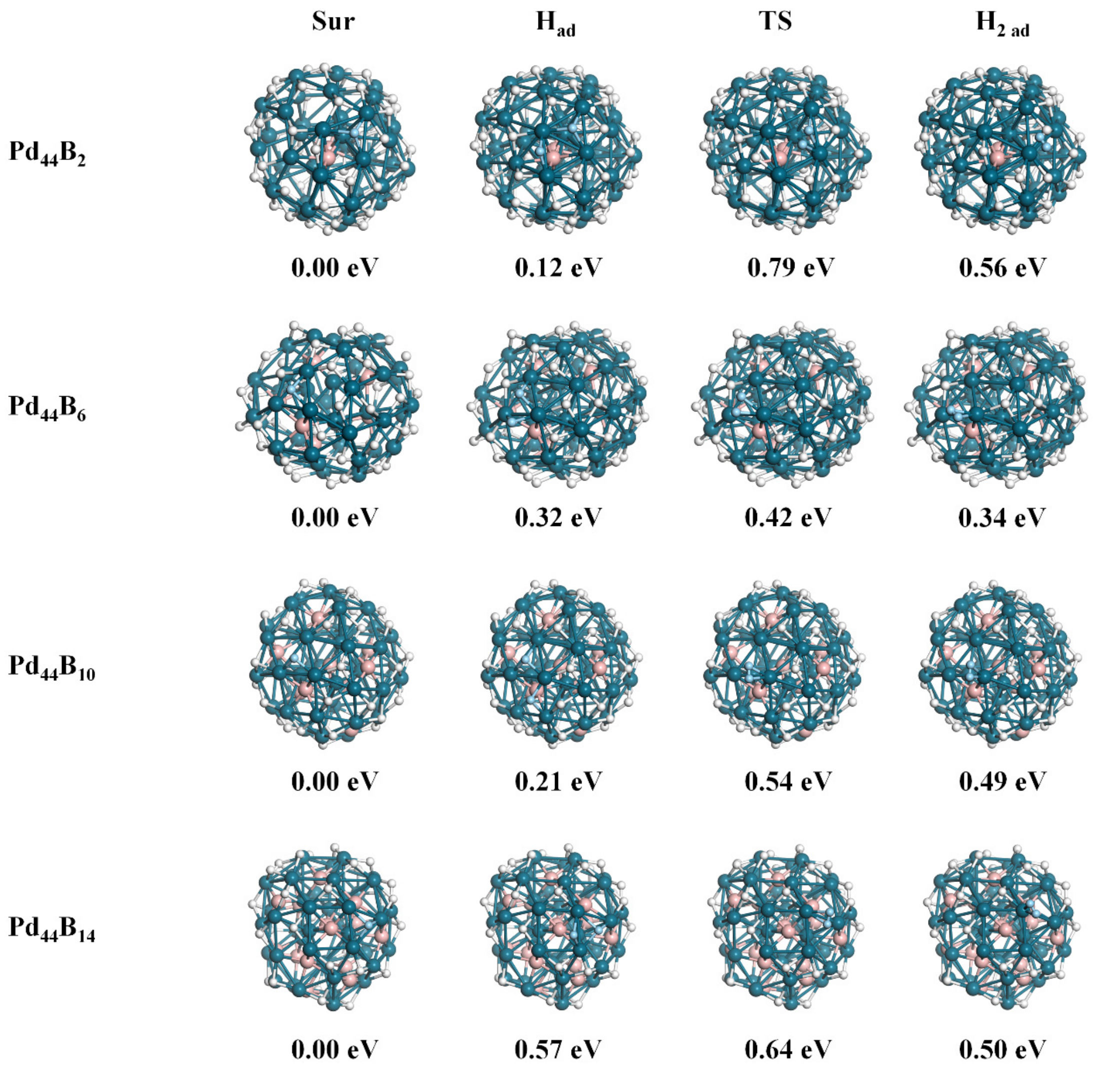

Figure S18. The structures of every step during HER pathway for $Pd_{19}B_y$ (y=2,6,10,14) clusters.

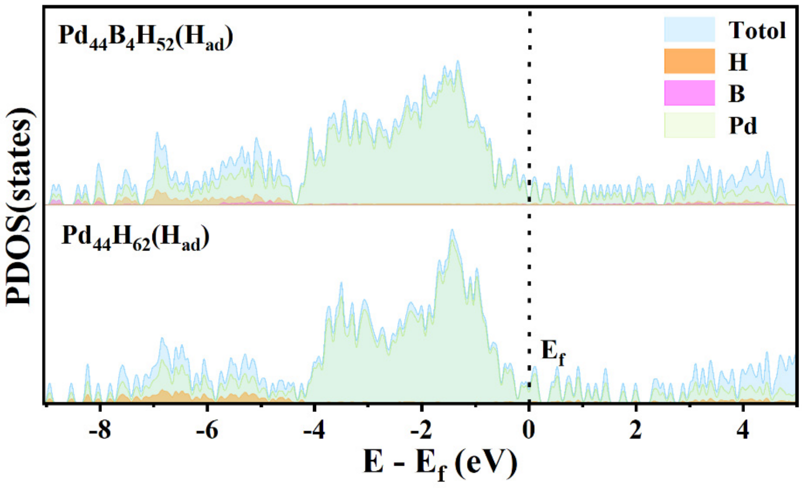

Figure S19. Partial wave density of state (PDOS) of $Pd_{44}B_4H_{52}$ and $Pd_{44}H_{62}$ (H_{ad} state for HER).
